# Randomized clinical trial of the effect of NovaMin and CPP-ACPF in combination with dental bleaching

**DOI:** 10.1590/1678-7757-2016-0408

**Published:** 2017

**Authors:** Larissa Dias ALEXANDRINO, Cristiane de Melo ALENCAR, Ana Daniela Silva da SILVEIRA, Eliane Bemerguy ALVES, Cecy Martins SILVA

**Affiliations:** 1 Universidade Federal do Pará, Belém, PA, Brasil.

**Keywords:** Dentin sensitivity, Tooth bleaching, Dentin desensitizing agents

## Abstract

**Objective:**

This randomized, controlled, double-blind clinical study evaluated the effect of calcium sodium phosphosilicate (NovaMin) and casein phosphopeptide-amorphous calcium phosphate with fluoride (CPP-ACPF) on the prevention of post-operative sensitivity and on the effects of clinical bleaching treatment.

**Material and Methods:**

Sixty volunteers were selected according to inclusion and exclusion criteria and were randomly assigned into three groups (n=20): CG (control group) patients, who were treated with 35% hydrogen peroxide; NOVAG (NovaMin group) patients, who were treated with 35% hydrogen peroxide followed by the application of NovaMin; and CPPG (CPP group) patients, who were treated with 35% hydrogen peroxide followed by the application of CPP-ACPF. Both bioactive agents were applied for five minutes. An evaporative stimulus associated with a modified visual scale was used to analyze sensitivity 24 hours after each bleaching session. The color evaluation was performed on the maxillary central incisors using a spectrophotometer. Associations between the intervention group, bleaching session, and reported sensitivity were tested using Chi-square partitioning.

**Results:**

Color change values (ΔE) were analyzed using analysis of variance (ANOVA). The significance level used for both tests was 5%. In the intragroup assessment, the Friedman test showed that only the CPP-ACPF group showed no statistically significant difference (p<0.05) between baseline and first bleaching session. In the intergroup assessment, the Kruskal–Wallis test showed that the CPPG had less postoperative sensitivity after the first session, when compared to the other groups (p<0.05). Color change analysis (ΔE) showed a significant difference between the means obtained in the different bleaching sessions in all groups (p<0.05).

**Conclusions:**

This study showed that the combination of CPP-ACPF with 35% hydrogen peroxide significantly reduced post-operative sensitivity in the first session, compared with the other evaluated treatments. The association of CPP-ACPF and NovaMin did not affect the color change induced by tooth bleaching.

## Introduction

Tooth bleaching has become a popular approach because it is conservative and capable of changing tooth color^26^. The use of highly concentrated agents is preferred by many dentists in clinical practice because it affords the professional greater control, ensures greater patient safety, and requires fewer applications^24^. Bleaching agents have an oxidizing action that leads to the formation of free radicals, reactive oxygen species, and hydrogen peroxide ions. These reactive molecules attack chromophores, causing their degradation and resulting in a bleaching effect^29^.

The most common adverse effect resulting from bleaching is dentinal sensitivity^5^, which is characterized by the manifestation of acute, short-term, or transient pain^16^. Moreover, it is currently believed that oxygen bubbles are formed in the dentinal tubules during the application of peroxides and that these bubbles can move the intratubular fluid and activate intradental nerves, causing post-operative sensitivity^7^. This hypothesis therefore suggests that sensitivity after bleaching is a consequence of peroxide penetration into the internal tooth structure, causing the direct activation of neuronal receptors^18^.

Although saliva has a known remineralizing action^9^, it alone may not be able to increase the levels of available calcium and phosphate in the oral cavity and prevent the incidence of sensitivity^27^. To minimize or eliminate post-operative sensitivity, fluorides and/or remineralizing solutions have been used to maintain a positive balance between demineralization and remineralization^3^. Bioactive agents containing calcium and phosphorus ions have thus been used to treat dentinal hypersensitivity^12^. In the presence of saliva and biofilm, these ions can occlude the dentinal tubules and reduce nerve-ending excitability without affecting the tooth bleaching process^25^.

Calcium sodium phosphosilicate (NovaMin) and casein phosphopeptide-amorphous calcium phosphate with fluoride (CPP-ACPF) are bioactive products that have been used to promote enamel remineralization^17^ ^,^ ^20^, and they were therefore combined with bleaching treatments in this study.

An inorganic bioactive glass, known commercially as NovaMin, has also been used for treating dental hypersensitivity and for enamel remineralization. This material was originally developed to act on bone regeneration because it has a ceramic structure containing amorphous calcium and sodium. NovaMin is therefore a highly water-reactive phosphosilicate composed of fine powder particles that can physically obstruct the dentinal tubules^11^.

NovaMin is composed of sodium, phosphate, calcium, and silica, and it quickly releases calcium, phosphorus, and sodium ions when forming the hydroxyapatite layer^10^. NovaMin forms an amorphous layer rich in the ionic compound a_2_+3PO^4-^, which is crystallized by the incorporation of OH^−^ and 3CO^2−^ anions present in the solution, thereby remineralizing the tissue^30^.

Despite promising results in other areas, it is believed that the use of remineralizing bioactive agents to accelerate the remineralization process may impair the enamel’s permeability and consequently impair the quality of the bleaching treatment. The penetration of reactive oxygen species and free radicals may be prevented by the deposition of ionic crystals on the tooth surface^6^.

The various commercial bioactive compounds vary widely in their clinical efficacy; thus, clinical studies are necessary to confirm the effectiveness of these substances when associated with bleaching.

This randomized clinical trial aimed to evaluate the effect of two bioactive agents, NovaMin and CPP-ACPF, used after tooth bleaching, on the prevention of post-operative sensitivity and on the quality of the bleaching treatment. The null hypotheses tested in this study were as follows: H01 – there is no difference in post-operative sensitivity between the tested groups (control, NovaMin, and CPP-ACPF) at the different evaluation times; H02 – there is no difference between the tested groups (control, NovaMin, and CPP-ACPF) regarding color change (ΔE) at the different evaluation times.

## Material and Methods

### Ethical aspects

The study followed the guidelines published by the Consolidated Standards of Reporting Trials – CONSORT^22^. The Research Ethics Committee of the Health Sciences Institute, Federal University of Para, reviewed and approved the study. The clinical trial number in the Brazilian Registry of Clinical Trials (REBEC) is U1111-1157-3105.

### Population and sample calculation

The software BioEstat^®^ (Civil Society Mamirauá, AM, Brazil) was used to calculate the sample size using data from a pilot study, which was conducted with ten volunteers and that followed the same procedures as this study. A statistical power of 80%, an α error of 5%, and a sample loss prediction of 20% at the end of the study were considered for sample size calculation. The sample calculated for this study was of 20 patients per group, totaling 60 patients.

### Study design

**Figure 1:**
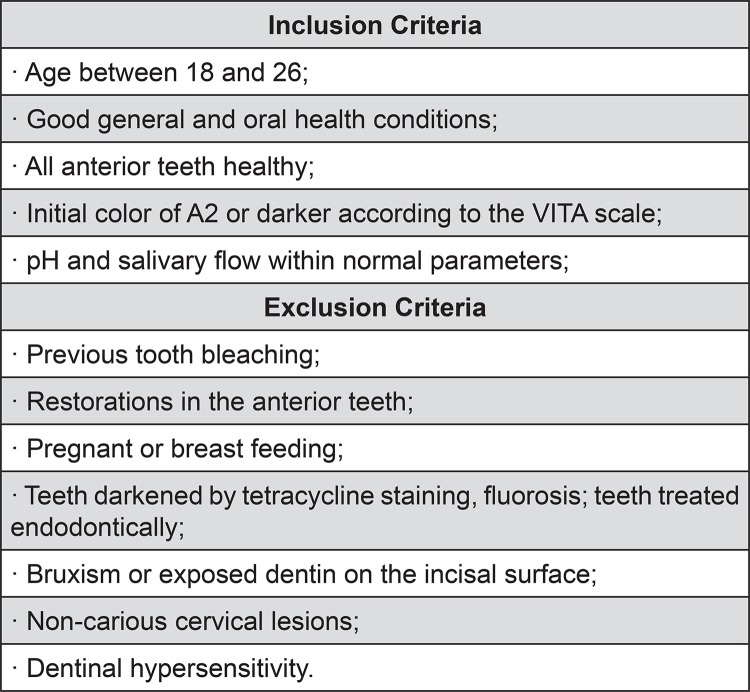
Inclusion and exclusion criteria

This randomized, controlled, double-blind study with three parallel arms evaluated comparatively the performance of bioactive agents recently proposed for reducing dentinal sensitivity after bleaching treatment. Sixty volunteers were selected according to the inclusion and exclusion criteria (Figure 1) and were divided into three groups by simple randomization (n=20). All groups underwent the same bleaching treatment. The gum tissue adjacent to the whitened teeth was isolated using a polymerized resinous gum barrier (Top Dan – FGM; Joinville, SC, Brazil). Thirty-five percent hydrogen peroxide (Whiteness HP – FGM; Joinville, SC, Brazil) was applied to the buccal surfaces of the incisors, canines, and premolars of both arches of the volunteers for 45 minutes. The bleaching treatment was performed over a total of three sessions, with an interval of seven days between them.

Patients in the experimental groups received an application of bioactive agents with a disposable applicator (Microbrush – Vigodent; Joinville, SC, Brazil) for five minutes on the bleached tooth surfaces after each bleaching session. The bioactive agents were later removed with water jets. Patients in the NOVAG (NovaMin group) were treated with NovaMin Repair and Protect (GSK Sensodyne; Brentford, Middlesex, United Kingdom), and patients in the CPPG (CPP group) were treated with CPP-ACPF (GC MI Paste Plus – Recaldent^®^; Hasunuma-Cho, Itabashi-Ku, Tokyo, Japan).

All patients received oral hygiene kits containing a toothbrush (Oral B Indicator; São Paulo, SP, Brazil) and toothpaste with 1,450 ppm fluoride (Colgate-Palmolive Company; São Paulo, SP, Brazil), along with instructions to use them three times per day.

The post-operative sensitivity evaluation was performed 24 hours after each bleaching session. An evaporative test was performed by applying air jets with a triple syringe (Dabi Atlante; Ribeirão Preto, SP, Brazil) to the labial surface of the bleached teeth. The volunteers received a modified visual scale (printed) containing facial expressions showing different levels of sensitivity and discomfort caused by the bleaching treatment (absent, mild, moderate, or severe).

The color evaluation was performed on each volunteer’s upper incisors using an Easyshade Advance spectrophotometer (Easyshade; Vita Zahnfabrik, Bad Säckingen, Germany) with the CIE L*a*b* system, where the color change values (ΔE) were obtained for each group of teeth using the following formula: ΔE={(ΔL)^2^+(Δa)^2^+(Δb)^2^}^1/2^, where ΔL*=L*−L*_0_; Δa*=a*−a*_0_; and Δb*=b*−b*_0_. The color evaluation was performed four times: before the bleaching treatment (baseline), and 24 hours after each of the three bleaching sessions.

To standardize the color measurement point on each tooth, a mold was made of each patient’s upper arch, and a plaster model was obtained to construct a polyethylene plate with the aid of a laminator. When the mold was ready, fixed reference points were established using a perforation in the central region of the labial surface of the maxillary central incisors of each model, with a size corresponding to the spectrophotometer tip^4^.

### Statistical analysis

The color change values (ΔE) for each group of studied teeth and the sensitivity reported by the volunteers were tabulated in an Excel spreadsheet (Microsoft Windows 2007) and analyzed using the program BioEstat^®^. The association between the intervention group, the bleaching session, and the sensitivity reported after 24 hours was tested by analysis of variance (Friedman or Kruskal–Wallis). The ΔE values obtained for each intervention group and each time point were analyzed using analysis of variance (ANOVA) followed by Tukey’s test. The significance level adopted in all analyses was 5%.

## Results

**Figure 2:**
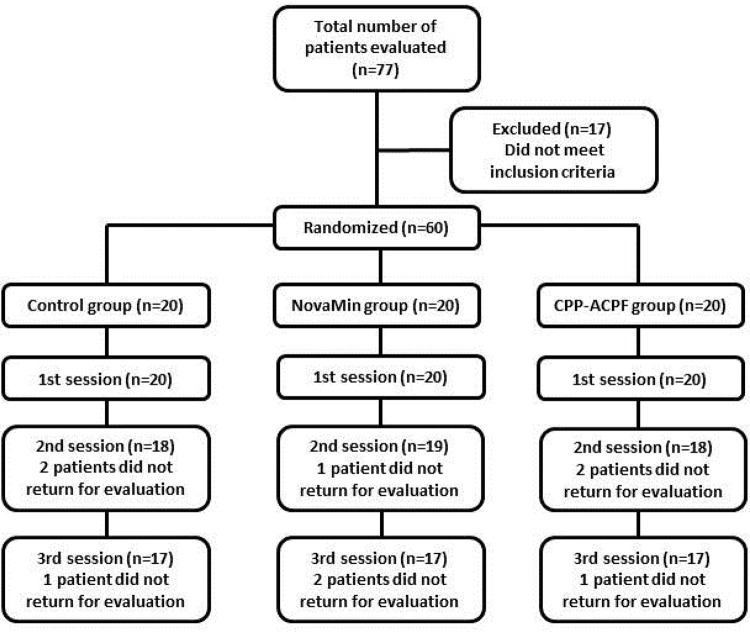
Schematic study design

Of the 60 randomized volunteers, 51 completed the treatment. Therefore, at the end of the study, 17 volunteers were evaluated in each experimental group (Figure 2). Table 1 shows the results for the evaluation of sensitivity reported by patients 24 hours after each bleaching session.

**Table 1:** Median (Md) and interquartile ranges (IQR) of sensitivity described by patients after bleaching treatment, according to experimental group and session

Time/	Group 1 (control)	Group 2 (NovaMin)	Group 3 (CPP-ACPF)
Groups	Md (±IQR)	Md (±IQR)	Md (±IQR)
Baseline	0.0 (±0.0)^A,a^	0.0 (±0.0)^A,a^	0.0 (±0.0)^A,a^
1^st^ session	1.0 (±0.0)^B,a^	1.0 (±0.0)^B,a^	0.5 (±1.0)^A,b^
2^nd^ session	1.0 (±0.0)^B,a^	1.0 (±0.0)^B,a^	1.0 (±0.0)^B,a^
3^rd^ session	1.0 (±1.0)^B,a^	1.0 (±1.0)^B,a^	1.0 (±0.0)^B,a^

Note^1^: Equal letters for similar values (p >0.05) and different letters statistically different values (p <0.05).

Note^2^:Capitalization is the comparison in the same column by Friedman test for comparisons intra groups; lower case letters representing the comparison on the same line by Kruskal-Wallis test for comparisons intergroup.

In the intragroup assessments, the Friedman test showed that only the CPP-ACPF group presented no statistically significant difference (p>0.05) between baseline and first bleaching session. In the intergroup assessments, the Kruskal–Wallis test showed that the CPP-ACPF group had less post-operative sensitivity after the first session, compared with the other groups (p<0.05).

The ΔE values for the upper incisors were used to compare the color changes between the experimental groups at each bleaching session (Table 2).

**Table 2:** Means, standard deviations, and 95% confidence intervals of ΔE values of the upper incisors, according to experimental group and session

Time/Groups	Group 1 (control)		Group 2 (NovaMin)		Group 3 (CPP-ACPF)	
	(±SD)	CI (95%)	(±SD)	CI (95%)	(±SD)	CI (95%)
1^st^ session	4.57 (±1.77)^A,a^	3.66-5.48	4.64 (±3.01)^A,a^	3.09-6.19	5.13 (±1.35)^A,a^	4.44-5.83
2^nd^ session	7.63 (±1.55)^B,a^	6.83-8.43	6.64 (±2.90)^B,a^	5.15-8.13	7.35 (±2.38)^B,a^	6.12-8.57
3^rd^ session	10.46 (±1.60)^C,a^	9.64-11.28	9.60 (±3.24)^C,a^	7.93-11.27	9.87 (±1.75)^C,a^	8.97-10.77
24 hours	9.84 (±2.60)^C,a^	8.50-11.18	10.10 (±3.98)^C,a^	8.06-12.15	9.85 (±2.17)^C,a^	8.73-10.96

Note^1^: Same letters for similar values (p> 0.05) and different letters for significantly different values (p<0.05).

Note^2^: Upper case letters represent comparisons between rows in the same column; lower case letters represent comparisons between columns in the same row by analysis of variance (ANOVA) followed by Tukey’s test.

The data analysis in Table 2 reveals that the variation in ΔE was homogeneous between the groups; there was no significant difference (p>0.05) when comparing the experimental groups at each treatment session. These data show that, under the conditions of this study, the groups treated with NovaMin and CPP-ACPF followed the same pattern of the control group, with ΔE increasing up to the third session and with color stabilization after this point.

In addition, when analyzing the groups individually, a clear increase was observed in the ΔE value at the end of the experiment in all tested groups. Both CG and CPPG groups showed an increase in ΔE after the second bleaching session (p<0.05); however, this color difference was only observed after the third bleaching session in the NOVAG.

## Discussion

Hydrogen peroxide at a 35% concentration has a high capacity of penetrating the enamel and dentin because of its low molecular weight (34.0147 g/mol), as well as its ability to denature protein, increase ionic movement, and stimulate nerve receptors. These factors together explain the high incidence of tooth sensitivity^2^.

Most post-bleaching sensitivity prevention treatments are based on the use of desensitizing agents, where the proposed mode of action is nerve inhibition and/or depolarization^23^. According to Paula, et al.^19^ (2013), the use of desensitizing agents has produced better results than the use of painkillers and anti-inflammatory agents. Furthermore, prior studies claim that bioactive products with tubular occlusion activity can preserve dental tissue by forming a protective layer over the enamel^14^.

The null hypothesis (H01) of this study was rejected because we observed that post-operative sensitivity was significantly reduced in the CPPG. The groups differed in tooth sensitivity 24 hours after the first bleaching session. Only the CPPG volunteers reported a lack of sensitivity, while most members of the CG and NOVAG presented mild pain, not differing significantly from each other. These results can be explained by the action of CPP-ACPF, which acts as a calcium and phosphate carrier that transports these minerals to the tooth surface, promoting their supersaturation. Additionally, the presence of casein in this compound stabilizes calcium phosphate by changing the chemical structure of the tooth surface and promoting remineralization^28^.

Reynolds, et al.^21^ (2001) report that CPP-ACP attaches easily to the tooth surface and to the plaque bacteria surrounding the tooth. Therefore, the CPP-ACP deposits are formed in close proximity to the tooth surface. These authors hypothesized that, in acidic conditions, CPP-ACP promotes the formation of calcium ions and free phosphates, substantially increasing calcium phosphate levels in the plaque. This effect would thus maintain a state of supersaturation that inhibits enamel demineralization and promotes remineralization, explaining the results of our study.

By contrast, NovaMin only reduced post-operative sensitivity in the first session. This short-term effect is due to the rapid precipitation capacity of calcium and phosphate ions, leading to a more immediate occlusion of dentinal tubules^1^. These glasses form an integrated bond with hard tissue through the formation of a carbonated hydroxyapatite layer on tissue surfaces following immersion in physiological solutions^13^. This hydroxyapatite layer, in turn, is generally thought to help protein binding, leading to the formation of a tight bond with the enamel^8^. Therefore, further studies must be conducted to evaluate the dissolution mechanism of NovaMin.

Instrumental evaluation is preferred to visual assessment, because the former provides the most practical and statistically reliable process, enabling analysis in small areas. Color evaluation using a spectrophotometer is performed under the International Commission on Illumination (CIE) standardization, in which the L*, a*, and b* coordinate values of each tooth, referring to black and white (luminosity), red-green and blue-yellow variations, respectively, are evaluated. These measurements thereby allow a total color variation to be calculated (ΔE). A ΔE variation of 3.3 to 3.7 is clinically apparent after tooth bleaching, which is in line with our results^15^.

The second null hypothesis (H02) was accepted based on the results of this color change evaluation method. The color evaluation results of this study showed that the color changes in the groups treated with NovaMin and CPP-ACPF followed a pattern similar to that observed in the CG, with an increase in ΔE up to the third session. These results show that the substances used to prevent sensitivity did not compromise tooth bleaching.

The CPP-ACP associated with 0.2% fluoride (900 ppm) may have promoted not only remineralization, but also the formation of a more homogeneous enamel layer, which is less permeable to pigments derived from the diet. By contrast, the high solubility of the ionic layer formed by the NovaMin bioactive likely compromised the longevity of the bleaching treatment in this experimental group.

The results suggest that, under the examined conditions, tooth bleaching with CPP-ACPF causes less sensitivity in treated teeth than a bleaching agent alone or a bleaching agent plus NovaMin. None of the bioactive agents affected the tooth bleaching process.

## Conclusion

The combination of CPP-ACPF with a bleaching treatment significantly reduced post-operative sensitivity in the first week, when compared with a bleaching treatment without a bioactive agent. The combination of NovaMin with bleaching treatment produced results similar to those obtained with bleaching treatment without a bioactive agent, and the association of either CPP-ACPF or NovaMin with 35% hydrogen peroxide did not affect the bleaching process.
